# Ifosfamide/etoposide alternating with high-dose methotrexate: evaluation of a chemotherapy regimen for poor-risk osteosarcoma

**DOI:** 10.1038/sj.bjc.6690187

**Published:** 1999-03

**Authors:** M P Michelagnoli, I J Lewis, H R Gattamaneni, C C Bailey, L S Lashford

**Affiliations:** Paediatric Haematology and Oncology Unit, St James University Hospital, Leeds, LS9 7TF, UK; Radiotherapy Department, The Christie Hospital, Manchester, M20 9BX, UK; Department of Experimental Haematology, The Patterson Laboratories, Manchester, M20 9BX, UK

**Keywords:** relapsed osteosarcoma, ifosfamide, etoposide, high-dose methotrexate

## Abstract

Fifteen patients with relapsed osteosarcoma were treated with an intensive combination chemotherapy schedule. Ifosfamide 2.5 g m^−2^ daily and etoposide 150 mg m^−2^ daily coincidentally for 3 days and high-dose methotrexate 8 g m^−2^ (with folinic acid rescue) on days 10–14 in a planned 21-day cycle. Feasibility, toxicity and response to this alternative combination for the treatment of relapsed osteosarcoma was assessed. There were 98 evaluable cycles for toxicity and tolerability. The majority of cycles were well tolerated. Haematological toxicity of grade 3/4 (common toxicity criteria) was seen in all courses. Renal tubular loss of electrolytes, particularly magnesium, occurred in 71% of cycles. Thirteen per cent of cycles were repeated within 21 days and 61% within 28 days. In the thirteen patients evaluable for response, a partial response rate of 31% was seen after two cycles. However, patients with stable disease continued on therapy, and an overall consequent response rate of 62% was observed. Four patients were alive with no evidence of disease at 8–74 months. Three are alive with disease (at 8–19 months). There were six deaths, all disease related. This regimen exhibits an encouraging response rate in a group of children with poor prognosis disease, with a tolerable toxicity profile. © 1999 Cancer Research Campaign


*British Journal of Cancer* (1999) **79**(7/8), 1174–1178
© 1999 Cancer Research Campaign
Article no. bjoc.1998.0187

### Ifosfamide/etoposide alternating with high-dose methotrexate: evaluation of a chemotherapy regimen for poor-risk osteosarcoma

**MP Michelagnoli[1], IJ Lewis[1], HR Gattamaneni[2], CC Bailey[1] and LS Lashford[3]**

[1]Paediatric Haematology and Oncology Unit, St James University Hospital, Leeds LS9 7TF, UK; [2]Radiotherapy Department, The Christie Hospital, Manchester M20 9BX, UK; [3]Department of Experimental Haematology, The Patterson Laboratories, Manchester M20 9BX; UK

**Summary** Fifteen patients with relapsed osteosarcoma were treated with an intensive combination chemotherapy schedule. Ifosfamide 2.5 g $m^{-2}$ daily and etoposide 150 mg $m^{-2}$ daily coincidentally for 3 days and high-dose methotrexate 8 g $m^{-2}$ (with folinic acid rescue) on days 10–14 in a planned 21-day cycle. Feasibility, toxicity and response to this alternative combination for the treatment of relapsed osteosarcoma was assessed. There were 98 evaluable cycles for toxicity and tolerability. The majority of cycles were well tolerated. Haematological toxicity of grade 3/4 (common toxicity criteria) was seen in all courses. Renal tubular loss of electrolytes, particularly magnesium, occurred in 71% of cycles. Thirteen per cent of cycles were repeated within 21 days and 61% within 28 days. In the thirteen patients evaluable for response, a partial response rate of 31% was seen after two cycles. However, patients with stable disease continued on therapy, and an overall consequent response rate of 62% was observed. Four patients were alive with no evidence of disease at 8–74 months. Three are alive with disease (at 8–19 months). There were six deaths, all disease related. This regimen exhibits an encouraging response rate in a group of children with poor prognosis disease, with a tolerable toxicity profile.

**Keywords:** relapsed osteosarcoma; ifosfamide; etoposide; high-dose methotrexate

The development of treatment for patients with non-metastatic osteosarcoma limb primaries over the past 20 years has led to impressive improvements in survival with reported long-term relapse-free survival rates of 55–76% (Link et al, 1990; Bramwell et al, 1992; Meyers et al, 1992; Bacci et al, 1993). There are, however, recognized groups of patients who continue to have a poor outcome. These include patients with unresectable primary disease; those with metastatic disease at presentation; and those who relapse either locally or with distant metastases. Long-term disease-free survival for the first two groups is approximately 0–20% (Schaller et al, 1982; Goorin et al, 1985; Parham et al, 1985; Cassano et al, 1991; Bacci et al, 1992), and 5-year survival after metastatic relapse has been reported as ranging from 17% to 50% with metastatectomy and/or chemotherapy (Goorin et al, 1984; Huth and Eilber, 1989; Solheim et al, 1992; Tabone et al, 1994; Saeter et al, 1995).

Single-agent and multiagent chemotherapeutic trials have demonstrated the efficacy of a number of agents in the treatment of osteosarcoma – including doxorubicin (Cores et al, 1972), cisplatin (Nitschke et al, 1978), methotrexate (Jaffe et al, 1973) and ifosfamide (Marti et al, 1985). More recent phase II studies reveal roles for other agents in combination, most notably etoposide (Kung et al, 1985; Grana et al, 1989).

Since 1983, first-line therapy in the UK for children with non-metastatic limb primaries has been based on cisplatin/doxorubicin combinations within a series of Medical Research Council trials (Bramwell et al, 1992; Ornadel et al, 1994). In an attempt to provide an intensive chemotherapeutic approach for those failing conventional first line therapy, we have developed a schedule whereby other active agents, ifosfamide, etoposide and high-dose methotrexate, could be incorporated in an intensive manner. Ifosfamide and etoposide combinations have been evaluated previously in patients with recurrent paediatric sarcomas (Miser et al, 1987; Goorin et al, 1994; Tabone et al, 1994). High-dose methotrexate has evidence of activity both as a single agent and within multiple agent trials (Grem et al, 1988; Rosen, 1993). Of importance, high-dose methotrexate with folinic acid rescue can be given to neutropenic patients, thereby allowing another agent to be delivered during the myelosuppressive phase of the chemotherapy cycle and thus providing a degree of treatment intensification. This strategy has been successfully used in the intensification of treatment of non-Hodgkin's lymphoma (Muller-Weihrich et al, 1985).

This report describes the feasibility, toxicity and early response data of this combination.

*Received 9 December 1997*
*Revised 30 June 1998*
*Accepted 30 July 1998*

*Correspondence to:* I Lewis, Paediatric Department of Oncology, St James' University Hospital Trust, Leeds LS9 7TF, UK

### METHODS AND PATIENTS

### Treatment

The protocol was planned to deliver ifosfamide 2.5 g $m^{-2}$ over 24 h with mesna uroprotection at the same dose on 3 consecutive days. Coincident infusions of etoposide 150 mg $m^{-2}$ daily for 3 days were administered over 4 h. High-dose methotrexate (8 g $m^{-2}$ according to age) was given over 24 h on days 10–14 of a planned 21-day cycle (10% of dose in the first hour and 90% of dose infused over next 23 h) with appropriate hydration. Folinic acid

**Table 1** Outcome of treatment with ifosfamide/etoposide alternating with high-dose methotrexate (IEM) related to primary treatment details in relapsed, evaluable patients

| Patient no. | First-line therapy | Age at dx relapse | Months from primary dx | Site of relapse | Assessment after two cycles | Best outcome of IEM | No. of courses at best assessment | Surgery | Outcome after surgery | Max no. of courses | Current status | Survival in months from relapse |
|---|---|---|---|---|---|---|---|---|---|---|---|---|
| 1 | Cisplatin/doxorubicin | 15 | 9 | Lung | SD | PR | 11 | Bilateral thoracotomies | CR | 11 | NED | 12 |
| 2 | Cisplatin/doxorubicin | 14 | 13 | Lung | SD | CR | 10 | – | – | 12 | NED | 74 |
| 3 | Cisplatin/doxorubicin | 12 | 19 | Lung | PR | PR | 2 | Thoracotomy | CR | 3 | DOD | 30 |
| 4 | Cisplatin/doxorubicin | 14 | 10 | Lung | PR | PR | 6 | Bilateral thoracotomies | CR | 7 | AWD | 8 |
| 5 | Cisplatin/doxorubicin | 15 | 19 | Lung | SD | SD | 4 | Bilateral thoracotomies | CR | 5 | NED | 8 |
| 6 | Cisplatin/doxorubicin | 15 | 16 | Lung | PR | PR | 2 | Thoracotomy | CR | 6 | NED | 8 |
| 7 | Cisplatin/doxorubicin | 10 | 8 | Lung | SD | SD | 4 | – | – | 6 | DOD | 11 |
| 8 | Cisplatin/doxorubicin | 16 | 11 | Lung | PR | PR | 2 | – | – | 6 | DOD | 13 |
| 9 | Cisplatin/doxorubicin | 8 | 6 | Lung | [a] | PD | 3 | – | – | 3 | DOD | 7 |
| 10 | Cisplatin/doxorubicin | 15 | 11 | Lung, bone | SD[a] | PR | 6 | Bilateral thoracotomies | PR | 11 | AWD | 19 |
| 11 | Cisplatin/doxorubicin | 15 | 12 | Lung, bone, CNS | SD | PR | 6 | – | – | 11 | DOD | 18 |
| 12 | EOI multidrug | 12 | 26 | Bone | SD | SD | 3 | Above knee amputation | – | 3 | DOD | 8 |
| 13 | Cisplatin/doxorubicin | 16 | 11 | Local | – | SD | 1.5 | – | – | 2 | AWD | 9 |

dx, diagnosis; NED, no evidence of disease radiologically; AWD, alive with disease; DOD, dead of disease. [a]Assessment after three courses to suit patient circumstances.

rescue was started 36 h after the start of the methotrexate infusion at a dose of 15 mg $m^{-2}$ every 3 h from hour 36 to hour 48, and then 15 mg $m^{-2}$ 6 hourly until the methotrexate level was below $1.0 \times 10^{-7}$ M with appropriate adjustments according to methotrexate level monitoring (minimum ten doses.) Second and subsequent cycle scheduling were dependent on regain of haematological function, namely neutrophils $> 1.0 \times 10^{-9}$ and platelets $> 100 \times 10^{-9}$. Dose reduction was driven by cytopenia (grade 4) causing major sepsis or delays to haematological recovery requiring treatment delay of greater than 1 week.

For clarification of description, one course of ifosfamide/etoposide plus one course of high-dose methotrexate constitutes one cycle of chemotherapy.

Tumour surgery was scheduled on an individual basis when response to chemotherapy appeared maximal.

### Patients

Sequential patients treated with the new combination schedule had relapsed either with recurrent local disease or distant metastatic disease. Appropriate local Ethical Committee approval was acquired in each of the participating centres.

### Assessment at entry

Disease assessment at entry included physical examination with measurements of the diameter of known disease when appropriate. Imaging studies included plain radiographs of the primary lesion, and computerized tomography (CT) and/or MRI scans of the primary, in all those who had not previously had amputation of the primary site. All had CT scans of the chest and radionuclide bone scans.

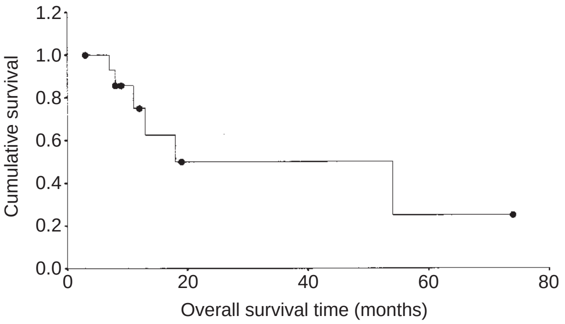

**Figure 1** Survival from commencement of combination regimen

Laboratory investigations included evaluation of the full blood count (FBC), serum electrolytes, urea and creatinine, liver function tests, calcium, magnesium and phosphate. The glomerular filtration rate was also assessed using [$^{99m}$Tc] DTPA (diethylene triamine penta-acetic acid).

### Assessment of response

Response of pulmonary metastases was assessed radiologically by CT scan and/or plain radiographs. Isotope bone scans were also performed to monitor bone metastases. Assessments were usually performed after two and six complete cycles. Definitions of response were as follows: complete response (CR), radiological disappearance of all evidence of tumour; partial response (PR), greater than or equal to 50% reduction radiologically in the tumour diameters at all sites; stable disease (SD), < 25% decrease in size

© Cancer Research Campaign 1999

of one or more lesions; and progressive disease (PD), progression of one or more of the tumours by greater than or equal to 25% of the diameter, or appearance of disease at a new site.

### Toxicity

Toxicity was assessed using the CTC (common toxicity criteria) (Franklin et al, 1994), with grade 3 indicating severe and grade 4 indicating unacceptable or life-threatening toxicity (excluding myelosuppression).

### RESULTS

### Patient characteristics

Thirteen patients with relapsed osteosarcoma were treated with the regimen and were evaluable for response. A further two patients had had tumour surgery at presentation and, consequently, were additionally evaluable for toxicity assessment.

There were seven males and eight females aged between 6 and 16 years (median 15 years). All fifteen patients had recurrent disease.

In those patients diagnosed with measurable relapsed disease, the time interval from initial presentation to diagnosis of recurrent disease ranged from 6 to 26 months. Lung metastases alone accounted for disease in nine patients. Three patients had bone secondaries, one in isolation, one in combination with both CNS and lung disease and the third patient had a combination of bone and lung recurrence. One child had a recurrence of his axial primary disease.

Twelve of these patients had been pretreated on cisplatin/doxorubicin schedules and one patient had received a multidrug regimen (methotrexate–vincristine–doxorubicin plus doxorubicin–cisplatin plus bleomycin–cyclophosphamide–dactinomycin) according to EOI guidelines (Souhami et al, 1997).

### Toxicity

There were 98 evaluable cycles, the majority of which were well tolerated. Myelosuppression of grade 3 or 4 was recorded in 80% of cycles and febrile neutropenic episodes requiring hospital admission occurred in 45% of cycles. Only three cycles caused severe sepsis (grade 4) necessitating subsequent dose reduction. Three patients required dose reduction of ifosfamide ± etoposide because of unacceptable haematological toxicity.

Significant renal toxicity was not common. Creatinine levels of greater than three times normal or a measured glomerular filtration rate (GFR) of $< 40$ ml $min^{-1}$ 1.73 $m^{-2}$ were seen in only four cycles and were reversible. Renal tubular loss of electrolytes, in particular magnesium, was common. Magnesium levels of $< 0.8$ mmol $l^{-1}$ (normal range 0.65–1.05 mmol $l^{-1}$) were recorded in 61% of cycles, but were correctable with oral supplementation. However, late-onset glomerular dysfunction (Prasad et al, 1996) was beyond the scope of this study in view of the limited follow-up interval.

Hepatotoxicity was limited to transient elevations of transaminases. Mucositis occurred in both the upper and lower GI tract in some patients, but was generally mild and short lived.

The consequent mean in-patient stay was 11.1 days per cycle (range 5–27 days). This figure includes both administration of chemotherapy and inpatient admissions with febrile neutropenic episodes.

An assessment of the tolerability of the first four cycles of treatment in terms of the ability to proceed on time to subsequent courses is conveyed by the finding that 13% of the first four cycles were administered within the prescribed 21 days, 33% within 25 days and 61% were given within 28 days.

### Responses

Thirteen patients with relapsed disease were evaluable for response (Table 1). The response rate after two cycles of chemotherapy was 31%. Patients with responsive or stable disease continued on therapy, achieving a best outcome after a further number of courses and thus giving an overall response rate of 62% (95% confidence interval 32–86%).

Eight out of 11 patients with pulmonary metastases achieved PR after 2–10 cycles (patient numbers 1–4, 6, 8, 11, 15). Two patients remained with stable disease (patients 5 and 7) and one had progressive disease (patient 9). Out of the group with partial response/stable disease, five were converted to CR with thoracotomy.

In the group with bony metastases, two patients out of three had a PR after 3–6 cycles. Both of these patients had combined lung and bone recurrence, PR was essentially judged on the basis of serial chest CT scans, both patients however demonstrated concomitant reductions in isotope bone scan activity. One patient had stable disease after two cycles, but refused further treatment because of side-effects. One child's disease progressed after three cycles of treatment.

Within the group of patients with relapsed disease, four patients are alive with no evidence of disease 8–74 months after entry into the study. Three patients are alive with radiological evidence of disease 8–19 months later. Six patients have died from their disease 7–30 months later. There were no toxic deaths.

Figure 1 details Kaplan–Meier analyses of overall survival from commencement of the chemotherapy regimen in evaluable patients. The median survival time was 18 months from commencement of the regimen, with 95% confidence intervals ranging from 0 to 54 months.

### DISCUSSION

The outlook for children and young adults with recurrence of osteosarcoma or primary metastatic disease remains poor. When the lung is the only site of disease recurrence, a surgical approach in which all resectable deposits are removed has been advocated as potentially curative, with a reported 5-year survival rate from first thoracotomy of 23–50% (Schaller et al, 1982; Meyer et al, 1987; Belli et al, 1989; Snyder et al, 1991; Skinner et al, 1992; Ward et al, 1994; Saeter et al, 1995; Han et al, 1996). Development of bony metastases carries a worse prognosis with a 0% 4-year survival rate (Ward et al, 1994).

The place of chemotherapy in the management of recurrent or advanced osteosarcoma is not of proven benefit, especially in those already heavily pretreated (Han et al, 1981; Meyer et al, 1987; Cassano et al, 1991). However, a number of regimens have been reported to have been used in this situation (Morgan et al, 1984; Solheim et al, 1992; Ward et al, 1994); including ifosfamide and etoposide in combination (Miser et al, 1987; Goorin et al, 1994; Tabone et al, 1994; Gentet et al, 1997).

Ifosfamide is considered by some authors to be one of the most effective treatments in osteosarcoma, with response rates recorded in the region of 67% (Chawla et al, 1990). Other phase II studies

 © Cancer Research Campaign 1999

confirm its efficacy as a single agent and in combination schedules (Marti et al, 1985; Harris et al, 1995). Etoposide has been used in a number of phase II trials in children with recurrent malignant solid tumours, and has shown a high proportion of complete and partial responses (Chard, 1979; O'Dwyer et al, 1985); however, single-agent usage in osteosarcoma has been disappointing. Support for its efficacy in combination with ifosfamide is provided by the results of the Rizzoli institute's second neoadjuvant study in which it was used as salvage therapy (Bacci et al, 1993), and the results of the Children's Cancer Group (Miser et al, 1987). The tolerability of this combination is aided by the fact that their non-haemopoiotic toxicities do not overlap.

There is a considerable body of evidence supporting the role of high-dose methotrexate in the treatment of osteosarcoma since its first use in the 1970s, both as a single agent and as part of multi-agent trials (Grem et al, 1988; Rosen, 1993). The advantages of adding high-dose methotrexate to an ifosfamide/etoposide combination include that, individually, it has an impressive single-agent profile; myelotoxic schedules can be resumed after only 1 week of administering HDMTX; and, properly administered, there is minimal toxicity. In addition, especially latterly, current MRC protocols for first-line treatment of osteosarcoma do not include high-dose methotrexate and therefore relapsing patients have not had prior experience of this drug.

The toxicity profile overall was tolerable. There was no treatment-related mortality. The renal tubular electrolyte loss related to ifosfamide chemotherapy required close monitoring and oral supplementation, but no patient required dose reduction because of renal complications. Haematological toxicity resulted in febrile neutropenia events that required inpatient stay and occasional dose-reduction consequently. But the majority of courses were well tolerated.

The results of this retrospective analysis suggest an encouraging response rate of 62%, in a group of children with poor prognosis disease. We have observed a delayed response rate to this regimen in patients who had stable disease at reassessment after two cycles. Patients with SD had radiological PR or CR by 6–10 cycles, indicating that classic phase 2 methodology of assessment after two cycles may not always be adequate. The number of patients evaluated are obviously too few to be considered statistically significant and the period of follow-up is limited. However, the regimen deserves consideration for an appropriate multi-institutional evaluation.

### REFERENCES

Bacci G, Picci P, Briccoli A, Avella M, Ferrari S, Femino FP, Monti C, Ruggieri P, Rizzente A and Casadei R (1992) Osteosarcoma of the extremity metastatic at presentation: results achieved in 26 patients treated with combined therapy (primary chemotherapy followed by simultaneous resection of the primary and metastatic lesions). *Tumori* **78**: 200–206

Bacci G, Picci P, Ferrari S, Ruggieri P, Casadei R, Tienghi A, Brach del Prever A, Gherlinzoni F, Mercuri M and Monti C (1993) Primary chemotherapy and delayed surgery for nonmetastatic osteosarcoma of the extremities. *Cancer* **72**: 3227–3238

Belli L, Scholl S, Livartowski A, Ashby M, Palangie T, Levasseur P and Pouillart P (1989) Resection of pulmonary metastases in osteosarcoma – a retrospective analysis of 44 patients. *Cancer* **63**: 2546–2550

Cassano W, Graham-Pole John and Dickson N (1991) Etoposide, cyclophosphamide, cisplatin and doxorubicin as neoadjuvant chemotherapy for osteosarcoma. *Cancer* **68**: 1899–1902

Chard RL Jr, Krivit W, Bleyer WA and Hammond D (1979) Phase II study of VP16-213 in childhood malignant disease: a Children's Cancer Study Group report. *Cancer Treat Rep* **63**: 1755–1759

Chawla SP, Rosen G, Lowenbraun S, Morton D and Eilber F (1990) Role of high dose ifosfamide in recurrent osteosarcoma. *Proc Am Soc Clin Oncol* **9**: A1201

Cores EP, Holland JF, Wang JJ and Sinks LF (1972) Doxorubicin in disseminated osteosarcoma. *JAMA* **221**: 1132–1138

Franklin HR, Simonetti GP, Dubbelman AC, ten Bokkel H, Huinink WW, Taal BG, Wigbout G, Mandjes IA, Dalesio OB and Aaronson NK (1994) Toxicity grading system. A comparison between the WHO scoring system and the CTC when used for nausea and vomiting. *Ann Oncol* **5**: 113–117

Gentet JC, Brunat-Mentigny M, Demaille MC, Pein F, Avet-Loiseau H, Berger C, De Lumley L, Pacquement H, Schmitt C, Sariban E, Pillon P, Bernard JL and Kalifa C (1997) Ifosfamide and etoposide in childhood osteosarcoma. A phase II study of the French Society of Paediatric Oncology. *Eur J Cancer* **33**: 232–237

Goorin AM, Delorey M, Lack EE, Gelber RD, Price K, Cassady JR, Levey R, Tapper D, Jaffe N, Link M and Abelson HT (1984) Prognostic significance of complete surgical resection of pulmonary metastases in patients with osteogenic sarcoma: analysis of 32 patients. *J Clin Oncol* **2**: 425–431

Goorin AM, Abelson HT and Frei E (1985) Osteosarcoma: fifteen years later. *N Engl J Med* **313**: 1637–1643

Goorin AM, Cantor A and Link MP for Pediatric Oncology Group, Chicago, IL, USA (1994) A phase I trial of etoposide (VP) and escalating doses of ifosfamide (IFOS) plus GCSF in recurrent pediatric sarcomas. *Proc Am Soc Clin Oncol* **13**: 425

Grana N, Graham-Pole J, Cassano WF, Gross S, Mehta P, Kedar A, Elfenbein G and Oblon D (1989) Etoposide (VP-16) infusion plus cyclophosphamide (CY) pulses: an effective combination for refractor cancer (abstract). *Proc Am Soc Clin Oncol* **8**: 300

Grem JL, King SA, Wittes RE and Leyland-Jones B (1988) The role of methotrexate in osteosarcoma. *J Natl Cancer Inst* **80**: 626–636

Han M-T, Telander RL, Pairolero PC, Payne WS, Gilchrist GS, Sim FH and Pritchard DJ (1981) Aggressive thoracotomy for pulmonary metastatic osteogenic sarcoma in children and young adolescents. *J Pediatr Surg* **16**: 928–933

Harris M, Cantor A, Goorin AM, Shochat S, Ayala AG, Ferguson WS, Holbrook T and Link MP (1995) Treatment of osteosarcoma with ifosfamide: comparison of response in pediatric patients with recurrent disease versus patients previously untreated: a Pediatric Oncology Group Study. *Med Pediatr Oncol* **24**: 87–92

Huth JF and Eilber FR (1989) Patterns of recurrence after resection of osteosarcoma of the extremity: strategies for treatment of metastases. *Arch Surg* **124**: 122–126

Jaffe N, Farber S, Paed D, Farber S, Traggis D, Geiser C, Kim BS, Das L, Frauenberger G, Djerassi I and Cassidy JR (1973) Favourable response of metastatic osteogenic sarcoma to pulse high dose methotrexate with citrovorum rescue and radiation therapy. *Cancer* **31**: 1367–1373

Kung F, Hayes A and Krischner J (1985) VP-16 in children with recurrent malignant solid tumours: a phase II study (abstract). *Proc Am Soc Clin Oncol* **4**: 235

Link M, Goorin A, Horowitz M, Meyer W, Belasco J, Baker A, Ayala A and Shuster J (1991) Adjuvant chemotherapy of high-grade osteosarcoma of the extremity. Updated results of the multi-institutional osteosarcoma study. *Clin Orthop* **270**: 8–14

Marti C, Kroner T, Remagen W, Berchtold W, Cserhati M and Varini M (1985) High dose ifosfamide in advanced osteosarcoma. *Cancer Treat Rep* **69**: 115–117

Meyer WH, Schell MJ, Kumar APM, Rao BN, Green AA, Champion J and Pratt CB (1987) Thoracotomy for pulmonary metastatic osteosarcoma: an analysis of prognostic indicators of survival. *Cancer* **59**: 374–379

Meyers PA, Heller G, Healey J, Huvos A, Lane J, Marcove R, Applewhite A, Vlamis V and Rosen G (1992) Chemotherapy for nonmetastatic osteogenic sarcoma: the Memorial Sloan–Kettering experience. *J Clin Oncol* **10**: 5–15

Miser JS, Kinsella TJ, Triche TJ, Tsokos M, Jarosinski P, Forquer R, Wesley R and Magrath I (1987) Ifosfamide with mesna uroprotection and etoposide: an effective regimen in the treatment of recurrent sarcomas and other tumors of children and young adults. *J Clin Oncol* **5**: 1191–1198

Morgan E, Baum E, Bleyer WA, Movassaghi N, Provisor A, Lampkin B, Lukens J, Griffin T, White H, Fryer C, Telander R, Sather H and Hammond D (1984) Treatment of patients with metastatic osteogenic sarcoma: a report from the childrens cancer study group. *Cancer Treat Rep* **68**: 661–664

Muller-Weihrich S, Henze G, Odenwald E and Riehm H (1985) BFM trials for childhood non-Hodgkin's lymphoma. In *Malignant Lymphomas and Hodgkins Disease: Experimental and Therapeutic Advances*. Cavalli F, Bonadonna G and Rozencweigh M (eds), pp. 633–642. Nijhoff: Boston

Nitschke R, Starling KA, Vats J and Bryan H (1978) Cis-diamine-dichloroplatinum (NSC-119875) in childhood malignancies. A Southwest Oncology Group Study. *Med Pediatr Oncol* **4**: 127–132

© Cancer Research Campaign 1999

O'Dwyer PJ, Leyland-Jones B, Alonso M, Marsoni S and Wittes RE (1985) Etoposide (VP16-213) current status of an active anticancer drug. *New Engl J Med* **312**: 692–700

Ornadel D, Souhami RL, Whelan J, Nooy M, Ruiz de Eliviva C, Pringle J, Lewis I, Steward WP, George R, Bridgewater J, Wierzbicki R and Craft AW (1994) Doxorubicin and cisplatin with granulocyte colony-stimulating factor as adjuvant chemotherapy for osteosarcoma: a phase II trial for the European Osteosarcoma Intergroup. *J Clin Oncol* **12**: 1842–1848

Parham DM, Pratt CB, Parvey LS, Webber BL and Champion J (1985) Childhood multifocal osteosarcoma: clinical and radiological correlates. *Cancer* **55**: 2653–2658

Prasad VK, Lewis IJ, Aparicio SR, Heney D, Hale JP, Bailey CC and Kinsey SE (1996) Progressive glomerular toxicity of ifosfamide in children. *Med Pediatr Oncol* **27**: 149–155

Rosen G (1993) An opinion supporting the role of high-dose methotrexate in the treatment of osteosarcoma. *Cancer Treat Res* **62**: 49–54

Saeter G, Hoie J, Stenwig AE, Johansson AK, Hannisdal E and Solheim OP (1995) Systemic relapse of patients with osteogenic sarcoma. Prognostic factors for long term survival. *Cancer* **75**: 1084–1093

Schaller RT, Haas J, Schaller J, Morgan A and Bleyer A (1982) Improved survival in children with osteosarcoma following resection of pulmonary metastases. *J Paediatr Surg* **17**: 546–550

Skinner KA, Eilber FR, Carmack Holmes E, Eckardt J and Rosen G (1992) Surgical treatment and chemotherapy for pulmonary metastases from osteosarcoma. *Arch Surg* **127**: 1065–1071

Snyder CL, Saltzman DA, Ferrell KL, Thompson RC and Leonard AS (1991) A new approach to the resection of pulmonary osteosarcoma metastases – results of aggressive metastatectomy. *Clin Orthop Related Res* **270**: 247–253

Solheim OP, Saeter G, Elomaa I and Alvegard TA (1992) The treatment of osteosarcoma: present trends. *Ann Oncol* **3**(suppl. 2): 7–11

Souhami RL, Craft AW, Van der Eijken JW, Nooji M, Spooner D, Bramwell VH, Wierzbicki R, Malcolm AJ, Kirkpatrick A, Uscinska BM, Van Glabbeke M and Machin D (1997) Randomised trial of two regimens of chemotherapy in operable osteosarcoma: a study of the European Osteosarcoma Intergroup. *Lancet* **350**: 911–917

Tabone M-D, Kalifa C, Rodary C, Raquin M, Valteau-Couanet D and Lemerle J (1994) Osteosarcoma recurrences in paediatric patients previously treated with intensive chemotherapy. *J Clin Oncol* **12**: 2614–2620

Ward W, Mikaelian K, Dorey F, Mirra J, Sassoon A, Holmes EC, Eilber FR and Eckardt JJ (1994) Pulmonary metastases of stage 11B extremity osteosarcoma and subsequent pulmonary metastases. *J Clin Oncol* **12**: 1849–1858

*© Cancer Research Campaign 1999*
